# Investigation of the CTLA-4–CD28 Axis in Oral Squamous Cell Carcinoma

**DOI:** 10.3390/jcm14145171

**Published:** 2025-07-21

**Authors:** Ferdinand Feldmeier, Manuel Weber, Franca Pacelli, Christoph Vogl, Jacek Glajzer, Leah Trumet, Mayte Buchbender, Carol Geppert, Marco Kesting, Jutta Ries

**Affiliations:** 1 Department of Oral and Cranio-Maxillofacial Surgery, Friedrich-Alexander-Universität Erlangen-Nürnberg (FAU), Glückstraße 11, 91054 Erlangen, Germany; ferdfeld@tutanota.com (F.F.); manuel.weber@uk-erlangen.de (M.W.); fvpacelli@gmail.com (F.P.); christoph.vogl@uk-erlangen.de (C.V.); jacek.glajzer@uk-erlangen.de (J.G.); mayte.buchbender@uk-erlangen.de (M.B.); marco.kesting@uk-erlangen.de (M.K.); 2 Deutsches Zentrum Immuntherapie (DZI) and Comprehensive Cancer Center Erlangen-EMN (CCC ER-EMN), Friedrich-Alexander-Universität Erlangen-Nürnberg (FAU), 91054 Erlangen, Germany; 3 Department of Operative Dentistry and Periodontology, Friedrich-Alexander-Universität Erlangen-Nürnberg (FAU), 91054 Erlangen, Germany; leah.trumet@uk-erlangen.de; 4 Institute of Pathology, Friedrich-Alexander University Erlangen-Nürnberg (FAU), 91054 Erlangen, Germany; carol.geppert@uk-erlangen.de

**Keywords:** immune checkpoints, CTLA-4, CD 28, CD80, CD86, oral squamous cell carcinoma, OSCC

## Abstract

**Background:** Oral squamous cell carcinoma (OSCC) is a common head and neck cancer with low survival rates, especially in advanced stages, despite improved therapies. New developments show that immune checkpoint inhibitors (ICIs) are promising treatment options. A better understanding of immune suppression in OSCC could enable new therapeutic approaches and effective ICI combinations. **Methods:** The aim of this cross-sectional study was to investigate the significance of the differential expression of cytotoxic T-lymphocyte-associated protein 4 (CTLA-4), CD28 and their ligands CD80 and CD86 for the diagnosis and treatment of OSCC. To this end, mRNA expression was analysed by RT-PCR and compared in 65 healthy oral mucosa samples (NOM) and 104 OSCC samples. **Results:** The expression of CTLA-4 (a soluble and membrane-bound isoform) was increased in OSCC by 1.72-fold (*p* = 0.004) and 6.88-fold (*p* < 0.001), respectively. There was no significant difference for CD28 (*p* = 0.283), nor for the soluble isoform of CD86 (*p* = 0.845). The membrane isoform of CD86 was increased in OSCC by a factor of 1.39 (*p* = 0.009) and CD80 by 6.11-fold (*p* < 0.001). **Conclusions:** The results show a significant association between CTLA-4, CD80 and membrane-bound CD86 expression and diagnosis. They could improve diagnostics in multi-marker approaches and serve as therapeutic targets for ICI strategies. In particular, the data indicate a stronger immunosuppressive role of CD80 compared to CD86 in a tumor tissue context, suggesting the exploration of anti-CTLA-4 and anti-CD80 antibody combinations in animal models.

## 1. Introduction

Oral squamous cell carcinoma (OSCC) is a common type of malignancy of the head and neck. In 2020, there were 377,713 cases of OSCC worldwide in accordance with data acquired by the Global Cancer Observatory (GCO). The GCO predicts not only a rise in the incidence of OSCC of around 40% by 2040, but also an increase in mortality. Patients with OSCC often experience a significant impact on their quality of life as the disease progresses due to disfigurement and functional impairment [1].

Despite the progress being made in established treatments, including surgery, radiotherapy and chemotherapy, the survival rates remain at approximately 50% [2]. Recently, immunotherapy has developed into an effective new treatment option [2]. One aspect of immunotherapy is the administration of immune checkpoint inhibitors (ICIs). Immune checkpoint proteins (ICP) are capable of regulating the patient’s immune response. The administration of ICIs enables the restoration of the inhibited immune function, which is often compromised in OSCC [3]. Initially approved only for advanced cases with recurrence or metastases, ICIs may now be administered for untreated OSCC as a neoadjuvant treatment option [2]. In particular, anti-PD-1 immunotherapy has supported long-term survival in a modest proportion of cases. In a randomized phase 3 study of a PD-1 inhibitor in head and neck squamous cell cancer (HNSCC), the estimated one-year overall survival came to 36% compared to 16.6% with the administration of systemic standard chemotherapy. However, only 13.3% of patients responded to the treatment [4].

The lack of responsiveness to ICIs can be classified into two main categories: primary and secondary resistance. Patients with primary resistance do not respond to treatment from the beginning, while patients with secondary resistance may respond to initial treatment but will eventually lose responsiveness [5]. The causes of secondary or acquired resistance are numerous and include defects in antigen presentation, tumour-mediated immune suppression and the upregulation of additional inhibitory ICPs [3]. Therefore, more targets applicable in immunotherapies have to be identified. A promising concept to maximize response rates includes combining multiple ICIs [6]. In hepatocellular carcinoma, the blockade of the PD-1/L1 complex is associated with upregulation of CTLA-4, suggesting that direct additional blockade may prevent further immune escape. Indeed, the combination of the ICIs anti-PD-1/L1 and anti-CTLA-4 theoretically offers several advantages over single therapy with anti-PD-1/L1, as it reduces T cell anergy and increases the production of inflammatory cytokines [7]. The combination of PD-1/PD-L1 inhibitors with other ICIs or treatments is currently being investigated as a treatment option for OSCC. However, combining PD-1/PD-L1 inhibitors and CTLA-4 inhibitors has not yet demonstrated any enhancement of efficacy in advanced OSCC [3].

In order to develop more effective combinations of ICIs, it is essential to gain a deeper insight into the underlying mechanism of immunosuppression in OSCC. Furthermore, a more profound comprehension of these mechanisms may facilitate the discovery of novel therapeutic targets. In this context, it is important to have an outline of the underlying physiological mechanism of our immune system. The present study concentrated on the signalling of the CD28/CTLA-4 axis, which is one mechanism by which the immune system regulates the amplitude of the immune response.

**Figure 1:**
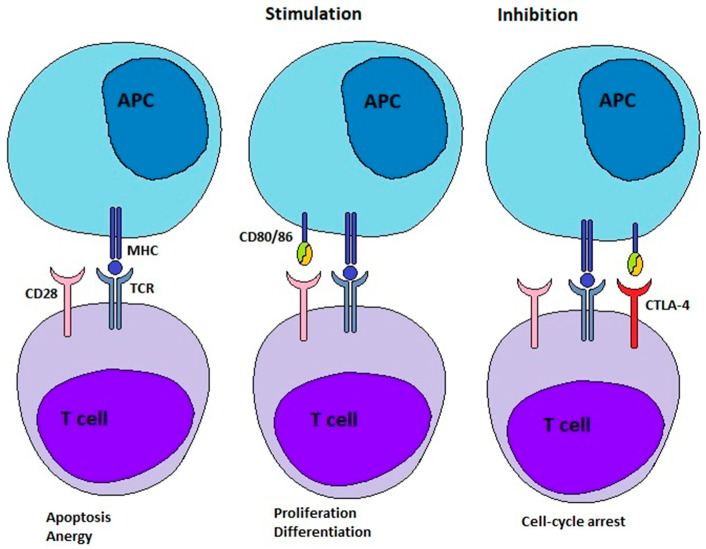
Comparison of the interaction between CTLA-4 and CD80/CD86 with that between CD28 and CD80/CD86. The outcome of the interaction between the T cell receptor (TCR) and the presented antigen is determined by the expression and interaction of additional receptors on the T cell. Without a second/costimulatory signal, the T cell becomes anergic or undergoes apoptosis. However, if CD28 recognises B7, the T cell becomes activated, which leads to proliferation and differentiation. CTLA-4 is then expressed on the T cell. Recognition of B7 by CTLA-4 results in cell cycle arrest, thereby preventing further T cell activation. Therefore, the effects of CD28 and CTLA-4 on the T cell are opposing. Although both receptors bind the same ligands, CTLA-4 has a higher affinity for B7 molecules. This figure is based on the presentation by Babamohamadi et al. [11].

Under normal immune-competent conditions, our immune system can detect and eliminate emerging tumour cells and stimulate anti-tumour immunity [8,9]. This concept is called cancer immunosurveillance [9]. To enable the process of immunoediting, T cells can recognize tumour antigens presented to their receptors (TCRs) by antigen-presenting cells (APCs) (Figure 1) [8]. However, a second/costimulatory signal is required to activate the T cell. One of several costimulatory signals is provided by the binding of CD28 molecules on T cells to their ligands expressed on APCs (Figure 1). These ligands on APCs are CD80 and CD86, also known as B-7 molecules. After successful activation, the expression of CTLA-4 is stimulated in the T cells. CTLA-4 is a coinhibitory ICP. It competes with CD28 for its ligands, the B-7 molecules. Because of the higher binding affinity of CTLA-4 to B-7 ligands, CTLA-4 can displace CD28. This leads to the attenuation and termination of T cell responses and the establishment of tolerance (Figure 1). The overexpression of CTLA-4 is a defining characteristic of exhausted and dysfunctional T cells. When CTLA-4 is deficient, individuals develop profound lymphoproliferation and systemic autoimmune disease. Thus, the ICP CTLA-4 plays a vital role in the adjustment of immune responses and the maintenance of homeostasis [8]. The binding of both ligands, CD80 and CD86, to CTLA-4 is followed by their destruction within the cell by transendocytosis. While binding with CD80, CTLA-4 remains ligand-bound, undergoes ubiquitylation, and ultimately degradation. Conversely, binding with CD86 leads to CTLA-4 detachment and recycling to the cell surface [10].

In this cross-sectional study, we analysed the mRNA expression of CD28 and CTLA-4 and their ligands CD80 and CD86 in NOM and OSCC tissues. A comparison was made between the expression levels in OSCC tissue and those in healthy oral mucosa tissue. The aim of this study was to ascertain the significance of differential expression of CTLA-4, CD28, CD80, and CD86 in the diagnosis and treatment of OSCC. We also wanted to elucidate the mechanisms by which OSCC can induce an immunosuppressive state that favours tumour growth and potentially enables the planning of new treatment strategies.

## 2. Materials and Methods

### 2.1. Study Design, Study Collective and Collection of Samples

The study uses a cross-sectional design. This type of design makes it possible to analyse correlations between variables at a fixed point in time.

In conducting this cross-sectional study, we endeavoured to adhere to the STROBE (Strengthening the Reporting of Observational Studies in Epidemiology) guidelines [12]. 

This analysis included a total of 169 subjects, divided into two groups: a test group (Oral squamous cell carcinoma (OSCC)) and a control group (Normal oral mucosa (NOM)). The OSCC group consisted of 104 patients with an initial diagnosis of oral squamous cell carcinoma and no prior treatment for study inclusion. The control group consisted of 65 normal oral mucosa samples (NOM) of healthy volunteers. The age and sex of all study participants were documented as shown in Table 1. OSCC and NOM groups matched regarding sex (*p* = 0.609), but not age (*p* < 0.001).

All samples were collected in the period from September 2017 to November 2021 at the Department of Oral and Cranio-Maxillofacial Surgery of the Friedrich-Alexander University Erlangen-Nürnberg.

The study was performed in accordance with the Declaration of Helsinki. A positive vote of the local ethics committee is present (ethics application number: 3962) and all patients consented to their participation in written form.

Healthy tissue (NOM group) was defined as non-inflamed and not malignant. All patients underwent minor maxillofacial surgery like dentoalveolar surgery or trauma surgery and were healthy regarding malignant diseases.

The following exclusion criteria were intended to ensure that the control group was healthy and had not undergone any treatment that modulates immune-related gene expression, such as corticosteroids or immunosuppressive agents, or suffered from systemic diseases involving the immune system.

Presence of inflammation in the oral mucosa.

Dysplasia or precancerous changes.

Periodontitis

Existing tumour diseases.

Immunological diseases or ongoing immunosuppressive therapies.

Participants under 18 years of age.

Additionally, exclusion criteria for tumour patients were pre-existing OSCC, immunological diseases or ongoing immunosuppressive therapies, and participants under 18 years of age.

OSCC tissue (OSCC group) was categorized regarding its grading (G1, G2, G3; differentiation status), the clinical UICC-stage (I-IV) and TNM classification according to the guidelines of the World Health Organization classification (2017) of tumours of the head and neck and the International Union Against Cancer [13,14,15].

Lymph node status was used to further separate OSCC patients by either the absence (N0) or the presence (N+) of lymph node metastases.

The clinical stages were grouped into early (stage I and II) and late (stage III and IV) stages and into small (T1 and T2) and large (T3 and T4) tumours depending on tumour size.

All parameters are given in Table 1.

### 2.2. Sampling of Tissue Specimens

Tumour specimens of the OSCC group were collected during tumour resection. The sample was divided. One part was sent to the pathology department to confirm the diagnosis. The remainder was made available for study purposes. Samples of the NOM group were acquired during minor routine surgeries. Each sample was immediately transferred into RNAlater (Qiagen, Hilden, Germany) and was fixed by incubation at 4 °C for at least 24 h. Until mRNA isolation, they were stored at −80 °C.

Samples of the OSCC group contained at least 70–80% malignant epithelial tissue.

### 2.3. Isolation of mRNA and RT qPCR Analysis

To isolate RNA from RNAlater samples, a tissue disruption using Precellys^®^ (Bertin Instruments Company, Montigny-le-Bretonneux, France) was performed, and total RNA was isolated using the Qiagen “miRNeasy mini-Kit” (Qiagen Company, Hilden, Germany) according to the manufacturer’s instructions.

To be able to assess the quality and quantity of the RNA samples, their concentrations were determined using the Nano-Drop 3.3 ND-1000 spectrophotometer (Thermo Fisher Scientific Inc., Waltham, MA, USA) and the respective ND-1000 software (version V3.7).

According to the manufacturer’s instructions, the “High-Capacity cDNA Reverse Transcription Kit” of Applied Biosystems™ (Thermo Fisher Scientific Inc., Waltham, MA, USA) was utilized for reverse transcription of RNA into cDNA.

For semi-quantitative analysis of CTLA-4, CD28, CD80 and CD86 expression gene-specific primers (Table 2) and the PowerSYBR^®^ Green PCR Master Mix of Applied Biosystems™ (Thermo Fisher Scientific Inc., Waltham, MA, USA) were employed.

For all primer pairs, the annealing temperature was 60 °C. The number of cycles amounted to 40.

The primers for CD28 and CD80 detect the mRNA corresponding to only one isoform. For CTLA-4 and CD86, two primer sets were applied for amplifying amplicons of different isoforms of the gene. The CTLA-4 gene encodes two isoforms. CTLA-4_var1 creates an amplicon of both isoforms (soluble and membrane-bound form). CTLA-4_var2 generates an amplicon exclusively specific to the membrane-bound form of CTLA-4. The CD86 gene encodes five isoforms. CD86_Iso1 created an amplicon of the isoforms 1, 2, 4, and 5 that are all membrane-bound. CD86_Iso3 generated an amplicon exclusively specific for the soluble isoform 3. 

The ΔCT method was used to normalize the collected data using GAPDH as an internal control.

Data acquisition and analysis was performed using QuantStudio 6 Pro from Applied Biosystems™ (Thermo Fisher Scientific Inc., Waltham, MA, USA).

### 2.4. Statistics

Data of gene expression originated from RT-qPCR and are presented as ΔCT values and mean ΔCT values ($\bar{\Delta CT}$).

Data were evaluated using the statistical software package SPSS 29 (SPSS Inc., Chicago, IL, USA). A *p*-value ≤ 0.05 was regarded as statistically significant.

As data were not normally distributed, only nonparametric tests were used in statistical analysis. 

A Mann–Whitney U test was calculated to check whether there are differences in gene expression between OSCC and NOM groups. To show the association of gene expression and clinical and histomorphological parameters the Kruskal–Wallis test was also utilized. The ΔΔCT-method (ΔΔCT_Gen1_ = average ΔCT_Gen1 OSCC group_ − average ΔCT_Gen1 NOM group_) was the basis of the relative quantification (RQ) of differences in gene expression between the OSCC group and the NOM group (RQ = Fold change (FC) = 2^−ΔΔCT^). RQ values were considered meaningfully increased superior to 2 and significantly decreased inferior to 0.5. 

To visualize the expression differences Box–Whisker plots were generated, displaying the median, interquartile range, and minimum and maximum values of the gene expression in the different groups. 

In order to prove statistical relevance receiver operating characteristic (ROC) curves were generated. This was achieved by plotting the sensitivity against the specificity (1-specificity). The area under the curve (AUC) is an indicator of efficacy that allows each marker to distinguish between the two groups. Following the establishment of statistical relevance, the optimal threshold respective cut-off point value (COP) for differentiating between OSCC and NOM group was determined by assessing the highest Youden indices for the specific gene. The COP was utilized to establish two categories per group, categorized as positive (below the COP) and negative for over-expression (above the COP). The Chi-square test was performed to clarify whether increased expression is associated with malignancy. 

A correlation analysis of mRNA expression of the different genes was carried out by Spearman’s correlation test. Spearman’s ρ was interpreted according to Cohen et al. (ρ = 0.1 weak, ρ = 0.3 moderate, ρ = 0.5 strong correlation) and was used to describe the strength of the correlation [16]. To visualize results, scatter plots were generated.

## 3. Results

### 3.1. Comparison of Tissue Gene Expression Between OSCC and NOM Group and Assessment of Statistical Relevance of Differential Expression 

There was a statistically significant difference in the expression of both CTLA-4_var1 (*p* = 0.004) and CTLA-4_var2 (*p* < 0.001) between OSCC and NOM. The expression levels of CTLA-4_var1 were increased by a factor of 1.72 in OSCC compared to NOM. CTLA-4_var2 expression levels were 6.88 times higher in OSCC compared to NOM.

For CD28, our results showed no statistical significance in expression levels between the two groups (*p* = 0.283). This was also shown for CD86_Iso3 (*p* = 0.845). However, the expression of both CD80 and CD86_Iso1 showed statistically significant differences in expression levels (*p*_CD80_ < 0.001 and *p*_CD86_Iso1_ = 0.009). The expression levels in OSCC were increased by a factor of 1.39 for CD86_Iso1 and 6.11 for CD80 compared to NOM.

**Figure 2:**
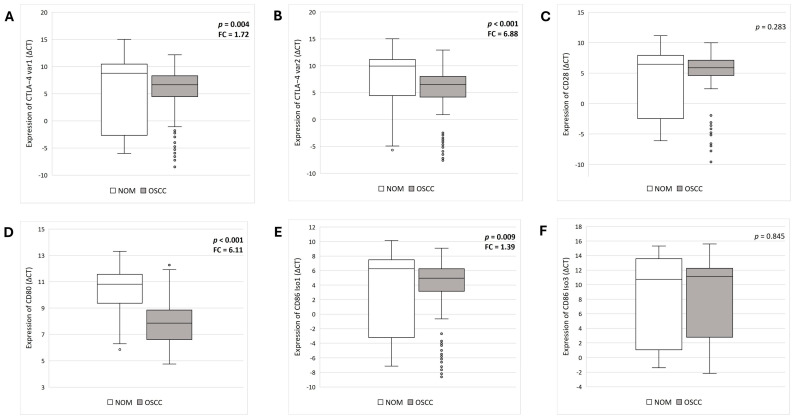
**Comparative analysis of the differential expression of CTLA-4_var1 and var2, CD28, CD80, CD86_Iso1 and Iso3 by RT-qPCR in OSCC vs. NOM**. The data are presented in the form of box plots. Expression levels are presented as median ΔCT values. Higher average ΔCT values indicate lower expression. *p*-values were calculated using Mann–Whitney U test. FC = Fold change = Relative change in expression level between the groups. (**A**) The expression of CTLA-4_var1 is increased in OSCC tissue. (**B**) The expression of CTLA-4_var2 is significantly increased in OSCC tissue. (**C**) No significant change in CD28 expression was observed between the groups. (**D**) The expression of CD80 is significantly increased in OSCC tissue. (**E**) The expression of CD86_Iso1 is significantly increased in OSCC tissue. (**F**) No notable alteration in CD86_Iso3 expression was observed between the groups.

The results are summarized in Table 3. Box plots were generated to visualize the results (Figure 2).

The AUC for significance of CTLA-4_var1 and var2 overexpression was 0.63 (*p* = 0.008) and 0.74 (*p* < 0.001), respectively. For variant 1, the sensitivity was 50.0% and the specificity was 85.1%. For variant 2, sensitivity was 60.3% and specificity was 91.2%. Applying the determined COP, 85.1% and 91.2% of OSCC patients showed overexpression of CTLA-4_var1 and var2, respectively, compared to only 50% and 39.7% of NOM samples. Chi-square test results showed that overexpression positivity was significantly associated with malignancy for both variants (*p*_CTLA-4_var1_ < 0.001 and *p*_CTLA-4_var2_ < 0.001). CTLA-4_var1 exhibited a sensitivity of 85.1% and a specificity of 50.0% for the detection of malignancy, while the PPV was 72.9% and the NPV was 68.1%. For variant 2, sensitivity was 91.2% and specificity was 60.3%, PPV was 78.8% and NPV was 80.9%.

Regarding the AUC for the significance of overexpression, the values for CD80 and CD86_Iso1 amounted to 0.87 for CD80 (*p* < 0.001) and 0.62 for CD86_Iso1 (*p* = 0.012). The results for CD80 showed a sensitivity of 86.5% and a specificity of 79.8%. For CD86_Iso1, the sensitivity reached 56.9%, the specificity of overexpression amounted to 73.1%. After using the determined COP to group the samples into negative/positive for overexpression, 79.8% and 72.1% of OSCC patients showed overexpression of CD80 and CD86_Iso1, respectively, compared to 13.5% and 43.1% of NOM samples. Chi-squared test results revealed that positivity for overexpression was significantly associated with malignancy for both genes (*p*_CD80_ < 0.001 and *p*_CD86_Iso1_ < 0.001). CD80 has a sensitivity of 79.8% and a specificity of 86.5% for detecting malignancy, with a PPV of 91.9% and NPV of 69.2%. CD86_Iso1 had a sensitivity of 72.1% and a specificity of 56.9% for detecting malignancy, with a PPV of 72.8% and NPV of 56.1%.

**Figure 3:**
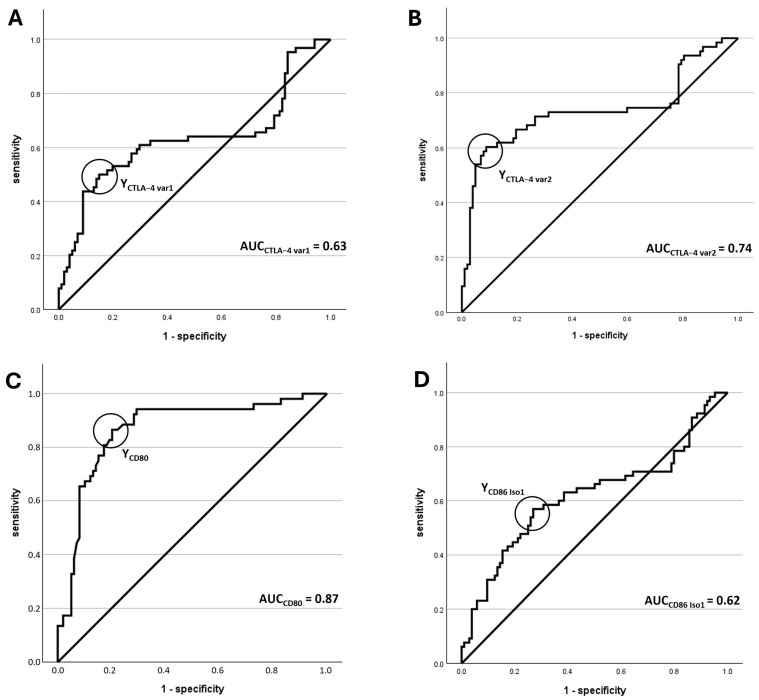
**Assessment of statistical relevance of differential expression in OSCC vs. NOM.** Receiver operating characteristic (ROC) curves were generated. The AUC (area under the curve) values demonstrate the significant association between the overexpression of the corresponding gene and malignant tissues. The circles indicate the highest Youden indices, which were used to determine the cut-off point (COP). (**A**) ROC of CTLA_var1. (**B**) ROC of CTLA_var2. (**C**) ROC of CD80. (**D**) ROC of CD86_Iso1.

**Figure 4:**
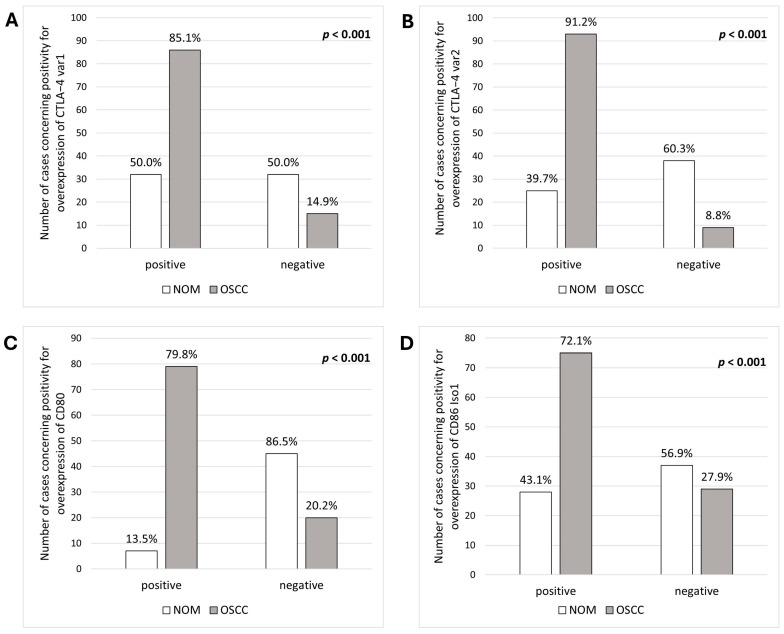
**Analysis of association between diagnosis and positivity of increased expression.** The OSCC and NOM groups were divided into positive and negative subgroups for overexpression based on the determined COPs. The association between malignancy and over expression = value under the COP was tested by the Chi-square test. The results demonstrated a significant association between malignancy and over expression of (**A**) CTLA-4_var1, (**B**) CTLA-4_var2, (**C**) CD80, and (**D**) CD86_Iso1.

A summary of statistically relevant results is provided in Table 4, with a visual representation in Figure 3 and Figure 4.

### 3.2. Correlation of Gene Expression Levels and Histomorphological and Clinical Parameters of OSSC Patients

No significant correlation between tumour size (CTLA-4_var1 (*p* = 0.103), CTLA-4_var2 (*p* = 0.363), CD28 (*p* = 0.647), CD80 (*p* = 0.103), CD86_Iso1 (*p* = 0.621), and CD86_Iso3 (*p* = 0.730)), lymph node status (CTLA-4_var1 (*p* = 0.167), CTLA-4_var2 (*p* = 0.745), CD28 (*p* = 0.988), CD80 (*p* = 0.124), CD86_Iso1 (*p* = 0.165), and CD86_Iso3 (*p* = 0.078)), grading (CTLA-4_var1 (*p* = 0.970), CTLA-4_var2 (*p* = 0.967), CD28 (*p* = 0.315), CD80 (*p* = 0.128), CD86_Iso1 (*p* = 0.187), and CD86_Iso3 (*p* = 0.259)), and recurrence (CTLA-4_var1 (*p* = 0.444), CTLA-4_var2 (*p* = 0.966), CD28 (*p* = 0.782), CD80 (*p* = 0.837), CD86_Iso1 (*p* = 0.399), and CD86_Iso3 (*p* = 0.534)) and the expression of the tested genes could be demonstrated by MWU or Kruskal–Wallis test. There was no correlation between UICC status and the expression of CTLA-4_var1 (*p* = 0.133), CTLA-4_var2 (*p* = 0.642), CD28 (*p* = 0.951), CD86_Iso1 (*p* = 0.090), and CD86_Iso3 (*p* = 0.217).

However, MWU results revealed a correlation between CD80 expression and late clinical status (*p* = 0.005). There was a 1.98-fold increase in CD80 expression levels in late UICC status compared to early UICC status. The AUC for significance of CD80 overexpression was 0.82 (*p* < 0.001). The sensitivity was 72.2%, while the specificity of CD80 overexpression was 80.0%.

**Figure 5:**
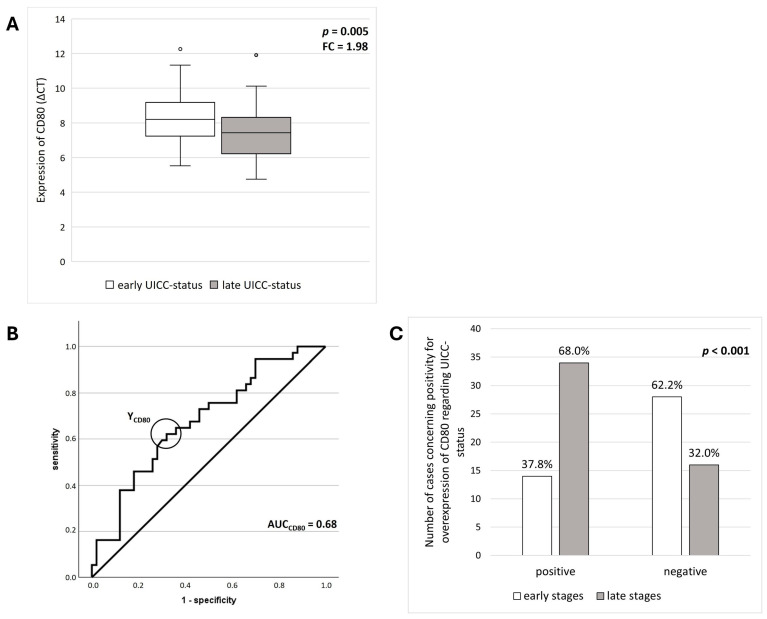
**Relationship between differential mRNA expression rates of CD80 and clinical stages of disease.** Statistical evaluation of differential mRNA expression level of CD80 in tissue specimens of OSCC between early and late clinical status. (**A**) Box plot showing the comparison of expression level between the groups. Higher average ΔCT values indicate lower expression. *p*-values were calculated using Mann–Whitney U test. FC = Fold change = Relative change in expression levels between the groups. The expression of CD80 is increased in OSCC tissue with late clinical stages. (**B**) A receiver operating characteristic (ROC) curves was generated. This was achieved by plotting the sensitivity against the specificity (1-specificity). The AUC (area under the curve) value substantiates the significant association between the overexpression of CD80 and late clinical stages. The circle indicates the highest Youden index, which was used to determine the ideal cut-off point (COP) for distinguishing between early and late stages. (**C**) The OSCC group was subdivided into positive and negative subgroups for overexpression based on the identified COP. The results generated by Chi-square test revealed a statistically significant association between late clinical stages and overexpression of CD80.

Applying the determined COP to group samples in positive/negative cases for overexpression, 68.0% of OSCC patients in late stages showed an overexpression of CD80 compared to 37.8% of patients in early stages. Chi-squared test results showed that positivity for overexpression was significantly associated with late-stage disease (*p* = 0.005). CD80 exhibited a sensitivity of 68.0% and a specificity of 62.2% for detecting late-stage disease, while the PPV was 70.8% and the NPV was 59.0%. The results for CD80 are presented in Figure 5.

The analysis of the localisation of squamous cell carcinomas revealed the following distributions: The highest prevalence was seen at the alveolar crest with 36.5%, followed by the tongue with 22.2%. The mucosa of the floor of the mouth (FOM) was affected in 27.9% of cases, while involvement of the buccal mucosa (BM) was comparatively low at 5.8%. Other localisations were the palate (3.8%) and the angle of the jaw (1.9%). The combination of FOM and tongue was observed in 3.8% of cases. Overall, the tumours were found to be relatively evenly distributed across the different sites, with the alveolar crest being the most frequently affected (Table 1).

There were no significant correlations between the expression rates of the ICPs examined and the respective localisations of the carcinomas. The Kruskal–Wallis test showed no significant *p*-values (p_CTLA-4_var1_ = 0.572, p_CTLA-4_var2_ = 0.717, p_CD28_ = 0.518, p_CD86_Iso1_ = 0.245, p_CD86_Iso3_ = 0.573, p_CD80_ = 0.654). These results indicate that the expression patterns of the analysed ICPs are not reliable markers for the specific localisation of oral squamous cell carcinomas.

### 3.3. Spearman Correlation Analysis of CTLA-4 and CD28 and Their Ligands CD80 and CD86

CTLA-4_var1 and CTLA-4_var2 mRNA expression data in tissue samples (OSCC and NOM) were strongly correlated with that of CD28 (Spearman’s ρ_CTLA-4_var1_ = 0.772, *p* < 0.001; Spearman’s ρ_CTLA-4_var2_ = 0.759, *p* < 0.001). While there was a strong correlation between expression of CD28 and both isoforms of CD86 (Spearman’s ρ_CD86_Iso1_ = 0.748, *p* < 0.001; Spearman’s ρ_CD86_Iso3_ = 0.626, *p* < 0.001), the correlation between expression of CD28 and CD80 was only moderate (Spearman’s ρ_CD80_ = 0.234, *p* = 0.008). A similar pattern was observed when looking at the correlation between CTLA-4 and its ligands CD80 and CD86. For both variants of CTLA-4, there was a strong correlation between both isoforms of CD86 (CTLA-4_var1: Spearman’s ρ_CD86_Iso1_ = 0.845, *p* < 0.001; Spearman’s ρ_CD86_Iso3_ = 0.648, *p* < 0.001; CTLA-4_var2: Spearman’s ρ_CD86_Iso1_ = 0.755, *p* < 0.001; Spearman’s ρ_CD86_Iso3_ = 0.514, *p* < 0.001). The correlation between CD80 and both variants of CTLA-4 was moderate (Spearman’s ρ_CTLA-4_var1_ = 0.388, *p* < 0.001; Spearman’s ρ_CTLA-4_var2_ = 0.463, *p* < 0.001). The correlation between the two isoforms of CD86 was strong (Spearman’s ρ = 0.733, *p* < 0.001), the correlation between CD80 and CD86_Iso1 was moderate (Spearman’s ρ = 0.352, *p* < 0.001). The correlation between CD80 and CD86_Iso3 was not statistically significant (Spearman’s ρ = 0.174, *p* = 0.062).

**Figure 6:**
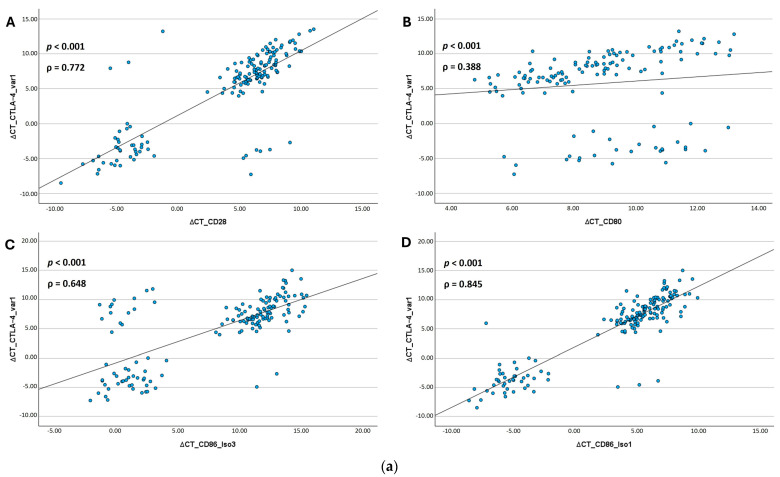

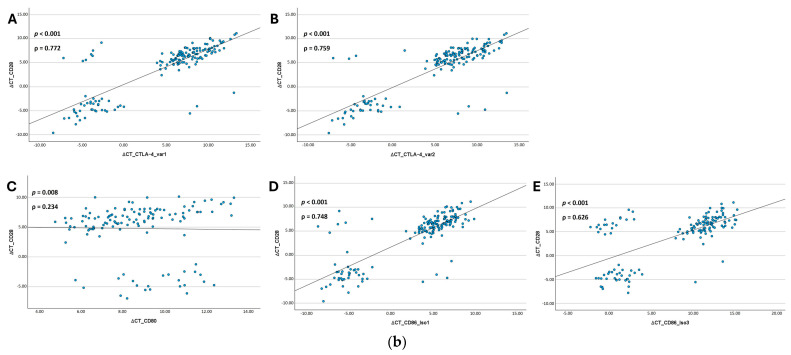
**Spearman correlation analysis of the expression levels of the investigated immunomodulators. (a) Spearman correlation analysis of CTLA-4 and CD28 and their ligands CD80 and CD86 in total sample with ΔCT of CTLA-4_var1 as dependent variable at the Y-axis**. The data is presented in the form of scatter plots. The expression levels are presented as ΔCT values. *p*-value was calculated by Spearman’s rho correlation test. ρ = Correlation coefficient. (**A**) A strong positive correlation was observed between the expression of CTLA-4_var1 and CD28. (**B**) A moderate positive correlation was observed between the expression of CTLA-4_var1 and CD80. (**C**) A strong positive correlation was observed between the expression of CTLA-4_var1 and CD86_Iso1. (**D**) A strong positive correlation was observed between the expression of CTLA-4_var1 and CD86_Iso3. **(b) Spearman correlation analysis of CTLA-4 and CD28 and their ligands CD80 and CD86 in total sample with ΔCT of CD28 as dependent variable at the Y-axis**. The data is presented in the form of scatter plots. The expression levels are presented as ΔCT values. *p*-value was calculated by Spearman’s rho correlation test. ρ = Correlation coefficient. A strong positive correlation was observed between the expression of CD28 and (**A**) CTLA-4_var1 and (**B**) CTLA-4_var2. (**C**) A moderate negative correlation was observed between the expression of CD28 and CD80. A strong positive correlation was observed between the expression of CD28 and (**D**) CD86_Iso1 and (**E**) CD86_Iso3.

All results are summarized in Table 5. Scatter plots were generated to visualize the results (Figure 6).

## 4. Discussion

This study represents foundational research regarding one aspect of the mechanisms of immunosuppression in OSCC. The aim of our study was to determine the importance of CTLA-4, CD28, CD80 and CD86 in the diagnosis and treatment of OSCC. 

As shown by string analysis [17], we expected that there would be an increase in the expression of checkpoint inhibitors, which would provide an explanation for tumour progression due to the immunosuppressive state observed in the tissue of OSCC.

The current study shows a significantly increased expression of ICP CTLA-4 as well as the ligands CD80 and CD86 in OSCC compared to NOM. CD80 and CD86, despite their capacity to stimulate the immune system when bound to CD28, have also been observed to contribute to immunosuppression through their interaction with CTLA-4 [8]. This is probably due to the higher binding affinity of the ligands to CTLA-4. Consequently, the increased binding to CTLA-4 results in a repressive rather than a stimulatory effect.

Our results indicate a significant association between the expression of these genes and the diagnosis of OSCC. Therefore, it is recommended to utilize these genes in a multi-marker approach to improve the detection of malignancies. 

We used two pairs of primers to detect the ICP CTLA-4. The primer pair for CTLA-4_var1 detects both the mRNA of the membrane-bound form and the soluble form of the protein. The primer pair for variant 2 is exclusively specific for the mRNA encoding the membrane-bound form of CTLA-4 (mCTLA-4). The expression of both variants was found to be significantly increased. The results demonstrate a significant disparity in fold change between the two variants of CTLA-4. One potential explanation is that the quantity of soluble CTLA-4 (sCTLA-4) constitutes the majority of total CTLA-4 present, and that it does not undergo a significant increase in OSCC tissue compared to healthy mucosa. Consequently, if both isoforms were detected simultaneously with CTLA-4_var1, our results were falsified due to the substantial proportion of sCTLA-4 that was also detected. This may indicate that sCTLA-4 has a limited role as an immunomodulator in OSCC, while the membrane-bound form has a more significant impact in this context. However, it may also be possible that sCTLA-4 suppresses the immune response more effectively than mCLTA-4. Therefore, the increase in sCTLA-4 expression does not need to be as pronounced as for the membrane-bound form to enable tumour cells to escape the immune system. A study investigating the functional capacity of sCTLA-4 for immunosuppression found that sCTLA-4 was able to reduce the proliferation of murine splenic CD8^+^ T cells in vitro and accelerate growth and experimental metastasis of murine tumours in vivo. In addition, in vivo results of the study suggest that a tumour releasing sCTLA-4 confers a survival advantage to malignant cells [18]. We suggest that future studies should also examine blood samples to ascertain whether the soluble form plays a greater role in this context. Additionally, for both variants, we found a highly significant association between positive CTLA-4 overexpression and malignancy. This means that a tissue sample can be assigned to the OSCC group or the NOM group, relying on CTLA-4 expression. 

This upregulation of CTLA-4 in the OSCC tissue samples was expected, as CTLA-4 is generally associated with immune tolerance [8]. Consequently, we hypothesize that CTLA-4 plays a pivotal role in immune tolerance in OSCC. CTLA-4’s primary function is to counterbalance the effect of CD28, the co-stimulatory receptor, in the early stages of T-cell activation, it also seems to play an important role in downregulating helper T cells [19]. CTLA-4 not only has a much higher binding affinity for the B-7 ligands than CD28 and may displace CD28 on the cell surface, but also exhibits the ability to capture and degrade B-7 by transendocytosis [8].

In a study conducted by Tekguc et al., it was demonstrated that Tregs expressing CTLA-4 constitutively depleted levels of CD80 and CD86 on antigen-presenting cells through transendocytosis. Moreover, CD80 can form cis-CD80/PD-L1 heterodimers, and the depletion of CD80 levels results in increased availability of PD-L1, which can then exert its inhibitory function on effector T cells [20]. In addition to complementing the immunosuppressive activity of Tregs and inhibiting effector T cells, CTLA-4 also appears to enhance the activity and proliferation of Tregs [19]. Therefore, the expression of CTLA-4 on Tregs can suppress the immune response through a variety of mechanisms [20]. 

In a study aimed to elucidate changes in gene expression in T cells during oral carcinogenesis, Xie et. al. showed that expression of CTLA-4 is upregulated during the development and progression of OSCC in mice [21]. Abdulkhaleq et al. concluded that a range of cancers can express CTLA-4. Their review included both studies that identified the protein and those that examined its expression at the mRNA level [22]. In the comprehensive analysis of CTLA-4 in the tumour immune microenvironment performed by Zhang et al., the authors also described a positive correlation between the expressions of CTLA-4 and CD28 and CD80, respectively, for most types of cancer [23]. The findings of our study are consistent with those of the previous research. Weber et al. performed an expression analysis of 21 immune regulatory checkpoint molecules in OSCC tissue [17]. In this study, OSCC tissue also showed an increased expression of CTLA-4. The correlation with the expression of CD80 was consistent with the findings of our study [17]. In addition, we observed a strong correlation between the ICP and its other ligand, CD86.

Our results show that there is an increased expression of CTLA-4 in OSCC tissue. This upregulation is thought to be a manifestation of T cell exhaustion. Previous studies have also suggested the expression of CTLA-4 as a marker of T-cell exhaustion and its severity [24]. Azharuddin et al. showed that in primary breast cancer, the exhaustive phenotype of T cells correlated with the expression of CTLA-4 [25].

For CD28, the current study did not find any statistical significance in expression levels between the two groups. However, Weber et al. showed an increased expression of CD28 in OSCC tissue compared to the healthy control group, even if at a low level [17]. Nonetheless, a recent study validates the findings of our study, demonstrating that there was no alteration in the expression of CD28 in the tissue of OSCC patients [26]. Our results suggest that CD28 plays a limited role in the immunosuppression mechanisms observed in OSCC tissue. Hence, based on our results, we would like to postulate that the expression level of CD28 does not have conclusive diagnostic value in OSCC.

The distinct functions of CD28 and CTLA-4 raise the question of how their ligands precisely control T cell responses. The ligands for CD28 and CTLA-4 are CD80 and CD86 [8]. The extant scientific literature describes a disparity in the interaction with their receptors between CD80 and CD86. Kennedy et al. postulate that the binding of CD80 to CTLA-4 results in transendocytosis of the CD80–CTLA-4 complex and ultimately to degradation in late endosomes/lysosomes. Whereas the binding of CD86 to CTLA-4 leads to transendocytosis as well, in this instance, the complex separates, the unmodified CTLA-4 is recycled back to the cell surface and CD86 is degraded. The study also reports a higher affinity of CD80 to CTLA-4 than CD86. It was observed that CD86 functions as a more effective CD28 ligand for the stimulation of both activated T cells and Tregs in the presence of elevated levels of CTLA-4 [10]. CD80+ T cells are reported to primarily display the defining characteristics of induced regulatory T cells. Conversely, CD86 is expressed on T cells that proliferate and manifest features characteristic of effector T cells [27,28].

In our study, expression of CD80 was significantly increased in OSCC samples compared to NOM. Additionally, CD80 expression was strongly associated with tumour diagnosis. Positivity of CD80 overexpression may be used to assign a tissue sample to one of the two groups. Moreover, OSCC specimens matching late UICC status showed significantly higher CD80 expression levels compared to OSCC specimens matching early UICC status. This result is supported by an expression analysis of patients suffering from HNSCC, 80% of the study participants exhibited overexpression of CD80, too [29].

For expression analysis of CD86, we used two different primer pairs to detect either mRNA of the membrane-bound forms or the soluble form of this protein. The primer pair CD86_Iso3 detected only mRNA for the soluble form (sCD86). Results presented no significant increase in expression of sCD86 in OSCC samples. The primer pair CD86_Iso1 was specific for identifying the mRNA of the membrane-bound forms of the protein (mCD86). Expression of mCD86 was found to be significantly elevated in OSCC tissue. However, the fold change was estimated to be a modest one. We observed that the positivity of overexpression of mCD86 was highly associated with the disease, meaning that specimens can be assigned to either the OSCC group or the NOM group, depending on mCD86 expression. 

A study examining the expression of B-7 molecules and their receptors in male patients diagnosed with late stages of OSCC also identified an increase in CD80 expression, as well as CD86 [26]. In a review about CD8 T cell exhaustion during chronic viral infection and cancer, the authors conclude that exhaustive T cells have high expression of inhibitory receptors and that the ligands for these receptors are upregulated in situations like chronic infections, autoimmune disorders, and cancer, where T cell exhaustion occurs [30]. Considering the evidence indicating that T cell exhaustion also occurs in OSCC tissue, our findings align with the conclusions of the aforementioned review. Our results demonstrated an upregulation of both the inhibitory receptor CTLA-4 and its ligands CD80 and CD86. Furthermore, a correlation was proven between the expression of the receptor and its ligand, as well as a correlation between CD80 and mCD86.

One objective of this study was to elucidate the mechanisms that drive the immune system to a more immunosuppressive state. As anticipated, an increase in the expression of the inhibitory receptor CTLA-4 was observed. Further investigation into this phenomenon suggests that, in addition to the receptor itself, its ligands should also be considered. The current study identified differences in the expression of B7 molecules in OSCC tissue, which may indicate varying interactions with their receptors. Experiments selectively blocking one of the two ligands, when both are expressed, reveal differences in their underlying effects. When blocking CD86, a more inhibited phenotype was observed [10]. This is supported by Manzotti et al. [31]. They postulated that in the presence of CD4+ CD25+ CTLA-4+ regulatory T cells, CD80 assumes the function of an inhibitory ligand. CD80 displayed the ability to inhibit the activation of resting human peripheral blood T cells by binding with CTLA-4, in contrast to CD86, which appears to facilitate T cell proliferation [31]. In a separate study, performed by Ke et. al., diabetic mice underwent transplantation of xeno-islets. Reportedly, the islets experienced a longer survival time when CD86-silenced dendritic cells with consistent CD80 expression were transferred [32].

The data suggest that CD80 is the ligand that modulates a more immunosuppressive response than CD86. Although Ke et al. [32] performed their experiments with CD86-silenced dendritic cells, we suggest that a parallel can be drawn. While both CD80 and membrane-bound CD86 expression were significantly increased in OSCC tissue in our study, CD80 expression was significantly higher. Another interesting perspective on this topic is provided by Sansom et al. [33]. The authors hypothesize that CD80 is critical in maintaining immune tolerance through its interaction with the inhibitory receptor CTLA-4, which functions to modulate the immune response and prevent autoimmunity [30]. During periods of inflammation, dendritic cells have been observed to upregulate CD86 in order to override the CD80-CTLA-4 inhibitory signals, thereby promoting immune activation [30]. This model highlights the delicate balance between immune tolerance and activation, with CD80 playing an essential role in maintaining tolerance, and dendritic cell signals tipping the balance towards activation in inflammatory contexts [30]. Studies show that CD80 acts as a functional ligand for CTLA-4, particularly in tumour models where CD80-transfected tumours exhibit reduced immunogenicity compared to those with CD86 [30]. In other experiments, CD80 has also been identified as a ligand responsible for the prolonged survival of vascularized heart grafts [30].

The fact that OSCC in late UICC stages shows a further increase in CD80 expression in our study suggests that CD80 plays an important role in immunosuppression and that higher expression levels promote further OSCC progression. Despite the large number of studies on CD86, there is no evidence that CD86 can send inhibitory signals via CTLA-4 [30]. Nevertheless, in the context of OSCC, our study demonstrates that both ligands exhibit increased expression and may be employed in a multi-marker approach for the detection of the disease.

The results of the current study may be promising for the identification of new targets for immunotherapy. We want to briefly describe the current state of scientific research regarding anti-CTLA-4 monoclonal antibodies (mAbs) and HNSCC.

ICIs prevent the CTLA-4 binding domain from interacting with its ligands, which in turn prevents the ICP from fulfilling its function. Thus, anti-CTLA-4 mAbs reactivate the inhibited immune system. Encouraging clinical results concerning treatments with these drugs in various forms of cancer endorse the idea that OSCC patients could benefit from the administration of ICIs targeting CTLA-4, too [8]. The only anti-CTLA-4 drugs approved by the FDA are ipilimumab and tremelimumab [3]. In non-small cell lung cancer (NSCLC), tumour cells expressed programmed death-ligand 1 (PD-L1) after treatment with anti-CTLA-4 antibody. PD-L1, in turn, not only favours tumour growth but also immune escape and drug resistance. Parviz et al. presented the idea that anti-CTLA-4 treatment, while aiding recovery in some cancer types, may induce mechanisms that mediate drug resistance and immune escape in other types of tumours [34]. A promising approach to address issues of this kind is the combination of multiple ICIs [6]. 

A recent study, performed by Ries et al., showed increased expression of BTLA, as well as PD-1 and CD96, in OSCC tissue. Hence, the authors list these ICPs as possible targets for immunotherapy. Results of this study also motivate testing the effectiveness of anti-BTLA therapy in combination with inhibition of PD-1 and/or CD96. BTLA resembles PD-1 and CTLA-4 in structure and function [35].

For HNSCC, most studies exploring combination therapies that include CTLA-4 inhibitors incorporate conventional treatment options, such as radiotherapy or the inhibition of the PD-1/PD-L1 axis. While anti-CTLA-4 mAbs are safe for the treatment of HNSCC, recent studies showed no significant clinical benefit from the inhibition of CTLA-4 [3]. In a study by Vackova et al. using a mouse model, the immunogenicity of tumour cells was investigated when CD80 expression was switched off. Interestingly, despite the stimulatory effect of CD80 binding to CD28 [8], it was found that deactivating CD80 reduces tumour growth and increases susceptibility to anti-CTLA-4 antibody treatment [36]. Consequently, it exerts a repressive rather than a stimulatory effect. In addition, the desired stimulatory effect of an ICI targeting CTLA-4 is reduced, as interaction between the actors is hampered by the presence of the CD80 ligand, which binds in the same area. Intercepting CD80 cancels out this effect and improves the efficacy of the ICI directed against CTLA-4 [36]. Although the study was conducted on mice, the findings indicate that anti-CTLA-4 treatment in humans may benefit from the administration of an additional dose of anti-CD80 antibodies. Our study demonstrated an upregulation of CTLA-4 suggesting that anti-CTLA-4 treatment may be a possible treatment option. Additionally, our study showed that CD80 is also upregulated in cases of OSCC. Hence, it may be postulated that treating patients with both anti-CTLA-4 and anti-CD80 antibodies may lead to a similar response rate in human tumours to that observed in mouse tumours.

Maurer et al. developed a CD80 variant fusion therapeutic (ALPN-202, davoceticept) that combines checkpoint inhibition with conditional CD28 co-stimulation. ALPN-202 shows a higher affinity for CD28 than the naturally occurring CD80. In addition, due to its increased affinity for the ligand, the novel therapeutic shows the ability to capture PD-L1 and inhibit CTLA-4 at the same time. In order to test the anti-tumour activity of ALPN-202, the authors used a murine solid tumour model of colorectal adenocarcinoma. The therapeutic proved to be more potent than WT CD80Fc or anti-human PD-L1 treatment in its ability to mediate anti-tumour activity in vivo [37]. Moreover, Kennedy et al. claim that their mouse model can accurately predict the immune response in humans [18].

It is reasonable to conclude from the evidence presented that combination therapy for OSCC with anti-CTLA-4 and anti-CD80 is a viable avenue for further investigation. We propose to investigate the effectiveness of anti-CTLA-4 mAbs in combination with anti-CD80 mAbs compared to administration of anti-CTLA-4 mAbs alone using the murine tumour model. 

As already mentioned above, CTLA-4 was shown to deplete CD80 levels, leading to reduced formation of cis-CD80/PD-L1 heterodimers, and increased availability of PD-L1, which can then exert its inhibitory function on effector T cells [20]. In this regard, administration of anti-CTLA-4 mAbs would increase the availability of CD80 to form heterodimers with PD-L1, thereby reducing the immunosuppressive effect of PD-L1 due to reduced availability. This would be a desired effect. Theoretically, the addition of anti-CD80 would abolish or counteract this effect. However, the underlying mechanisms are likely to be more complex. Nevertheless, the novel fusion therapeutic, ALPN-202, which captures PD-L1 in combination with CTLA-4 inhibition and CD28 costimulation [37], would be an obvious alternative to the classical anti-CTLA-4 mAb in this context.

### Shortcomings of the Study

In considering and interpreting the results, it is important to recognize that the sample size of this study should be increased. Another limitation we must point out is the presence of many outliers observed in the OSCC group for the results related to CTLA-4_var1, CTLA-4_var2, CD28, and CD86_Iso1. We are reasonably confident that measurement errors or instrumentation issues are not responsible for this. At this point, given the modest sample size, it remains uncertain whether this phenomenon is a manifestation of natural variability or a heavy-tailed or skewed distribution. Alternatively, it is plausible that certain phenotypes of OSCC exhibit elevated levels of expression for the aforementioned genes in comparison to the majority of OSCC cases. The decision was made to refrain from further data transformation or exclusion of outliers based on predetermined criteria, with the objective of avoiding the introduction of additional assumptions or biases. It is also essential to acknowledge the significant age disparity between the OSCC and NOM groups. Future researchers may consider additional strategies to address outliers and age discrepancy, particularly if the research is more focused on the specific relationship between age and gene expression in OSCC. However, the current statistical approach was deemed appropriate, given the characteristics of the data and the research objective. It must be acknowledged that the results of this study are regarded as preliminary. In order to establish the reliability of these results, further investigation is necessary.

Despite the valuable insights gained from the study, the results should be interpreted with the further limitation in mind. In particular, smoking habits, alcohol consumption and tumor location could affect comparability. However, these factors were not systematically recorded or analysed in the current study, as the focus was on using ICP expression to support diagnosis, prognosis and therapy development. For future research, we recommend taking these influencing factors into greater consideration to improve the results’ validity and transferability.

## 5. Conclusions

This analysis of human OSCC tissue samples revealed a significant upregulation of the expression of CTLA-4 and its ligand CD80 at the mRNA level. Expression analysis of the genes may be employed in a multi-marker approach to facilitate the detection of the disease. An analysis of the differential expression of B-7 molecules suggests that CD80 may function as the ligand responsible for inducing a shift towards a more immunosuppressive state in OSCC.

Immunotherapy has become a cornerstone of the battle against cancer. To increase the response rate, a combination of multiple ICIs may be administered. Although anti-CTLA-4 treatment has yielded encouraging outcomes in clinical trials in some forms of cancer, recent studies have demonstrated that it offers no clinical benefit in the context of OSCC, even when combining CTLA-4 inhibitors with other ICIs. Despite significant advances in the understanding of the CD28 and CTLA-4 receptors, knowledge of the two ligands lags behind. A comprehensive understanding of these differences is imperative to elucidate the regulatory mechanisms governing CD28 and CTLA-4 in T cell activation/inhibition. There is evidence that CD80 is the main ligand for CTLA-4. In this context, we propose to investigate the efficacy of anti-CTLA-4 mAbs combined with anti-CD80 mAbs using a murine tumour model.

However, it should be emphasised that the present results are exploratory and require validation in external cohorts and clinical studies.

## Figures and Tables

**Table 1:** **Demographic and clinical data of the study collective.** Staging parameters (T-, N-status, grading, clinical UICC-status) and occurrence of recurrence are shown. * Prognostic parameters are grouped: Tumour size (T1/T2 = small ones; T3/T4 = large ones), N-status (N0 = without lymph node involvement; N1 = lymph node involvement), UICC status (early = stage 1 and 2; late = stage 3 and 4), BM = Bottom of the mouth, FOM = Floor of the Mouth).

		Patients (OSCC)		Healthy Individuals (NOM)	
		N	% of Cases	N	% of Cases
**Number of cases**		104		65	
**Sex (*p* = 0.609)**	**Female**	35	33.7	26	40.0
	**Male**	71	68.3	39	60.0
**Mean age ± SD** **(*p* < 0.001)**		63.08 ± 12.17		54.01 ± 20.09	
**Range of age**		31–93 years		14–88 years	
			**Valid cases %**		
Tumour status *	**T1/T2**	62	63.3		
	**T3/T4**	36	36.7		
	**Unknown**	6			
N-status *	**N0**	54	54.0		
	**N+**	46	46.0		
	**Unknown**	4			
**Grading**	**G1**	11	10.9		
	**G2**	49	48.5		
	**G3**	41	40.6		
	**Unknown**	3			
**UICC status**	**Early**	40	42.6		
	**Late**	54	57.4		
	**Unknown**	10			
**Recurrence**	**R0**	67	70.5		
	**R1**	28	29.5		
	**Unknown**	9			
**Localisation**	**BM**	6	5.8		
	**FOM**	29	27.9		
	**Tongue**	21	20.2		
	**Alveolar crest**	38	36.5		
	**FOM/tongue**	4	3.8		
	**Palate**	4	3.8		
	**Jaw angle**	2	1.9		

**Table 2:** **Primer sequences for RT qPCR expression analysis.** For all primer pairs the annealing temperature is 60 °C. The number of cycles comes to 40. The CTLA-4 gene encodes two isoforms. CTLA-4_var1 creates an amplicon of both isoforms (soluble and membrane-bound form). CTLA-4_var2 produces an amplicon specific to the membrane-bound form of CTLA-4 only. The CD86 gene encodes five isoforms. CD86_Iso1 creates an amplicon of the membrane-bound isoforms 1, 2, 4, and 5. CD86_Iso3 generates an amplicon specific for the soluble isoform 3.

		*ACC*	*Forward Primer*	*Reverse Primer*
**CTLA-4**	**Var1**	NM_005214.5 NM_001037631.3	CCGTGCCCAGATTCTGACTT	GGGCTTCTTTTCTTTAGCATTTTGC
	**Var2**	NM_005214.5	TCCCTGTCTTCTGCAAAGCAAT	TCACACACAAAGCTGGCGAT
**CD28**		NM_006139.4	GTTGGTGGAGTCCTGGCTTG	GTAGTCACTGTGCAGGAGCC
**CD80**		NM_005191.4	GCAGGGAACATCACCATCCAA	CCACTTCTTTCACTTCCTTGGTC
**CD86**	**Iso1**	NM_175862.5 NM_006889.4 NM_001206924.1 NM_001206925.1	AAATGTGGAACCAACACAATGGAG	AAAACACGCTGGGCTTCATC
	**Iso3**	NM_176892.1	ACCTTTCTCTATAGGAACCAACACA	AAAACACGCTGGGCTTCATC

**Table 3:** **Expression analysis by RT qPCR.** Comparison of gene expression levels between OSCC and NOM groups and statistical evaluation of the differential mRNA expression rates of CTLA-4_var1, CTLA-4_var2, CD28, CD80, CD86_Iso1, and CD86_Iso3. Determination of statistical significance of the expression differences. The transcripts of CTLA-4, as well as those of CD80 and CD86_Iso1, were found to be significantly elevated in OSCC tissue. However, only CTLA-4_var2 and CD80 demonstrated a notable alteration in expression levels, as indicated by a fold change (FC) exceeding 2. Higher average ∆CT values indicate lower expression. N = number of cases, SD = Standard deviation, FC = Fold change = Relative change in expression level between the groups, MWU = Mann–Whitney U test, ROC = Receiver operating characteristic, AUC = Area under the curve, Y = Youden index, COP = Cut-off point given as ∆CT value determined by the highest Youden index, SEN = Sensitivity and SPE = Specificity for distinction between OSCC and NOM group.

.		N	Mean ∆CT Value	SD	FC	*p*-Value MWU	*p*-Value ROC	AUC Value	Y	COP	SEN %	SPE %
**CTLA-4_var1**	**OSCC**	101	4.87	5.33	**1.72**	**0.004**	0.008	0.63	0.35	8.81	50.0	85.1
	**NOM**	64	5.66	6.50								
**CTLA-4_var2**	**OSCC**	102	4.61	5.22	**6.88**	**0.001**	0.001	0.74	0.52	9.14	60.3	91.2
	**NOM**	63	7.39	5.78								
**CD28**	**OSCC**	101	4.12	4.84	0.96	0.283						
	**NOM**	61	4.06	5.50								
**CD80**	**OSCC**	99	7.92	1.67	**6.11**	**0.001**	0.001	0.87	0.66	9.04	86.5	79.8
	**NOM**	52	10.53	1.65								
**CD86_Iso1**	**OSCC**	104	3.10	4.85	**1.39**	**0.009**	0.012	0.62	0.30	5.84	56.9	73.1
	**NOM**	65	3.58	5.57								
**CD86_Iso3**	**OSCC**	95	8.71	5.15	0.47	0.845						
	**NOM**	52	7.62	6.30								

**Table 4:** **Analysis of association between diagnosis and positivity of increased expression.** Positivity of increased expression means a ∆CT value lower than the COP in a specific tissue sample. Overexpression of both variants of CTLA-4, CD80, and CD86_Iso1 was found to be significantly associated with malignancy, with a high level of statistical significance. *p*-value provided by the Chi-squared test, COP = Cut-off point given as a ∆CT value determined by the highest Youden index, N = number of cases, SEN = Sensitivity and SPE = Specificity for diagnostic use between OSCC and NOM group, PPV = Positive predictive value, NPV = Negative predictive value.

		*p*-Value	COP	N	N Pos (%)	N Neg (%)	SEN %	SPE %	PPV %	NPV %
**CTLA-4_var1**	**OSCC**	0.001	8.81	101	86 (85.1)	15 (14.9)	85.1	50.0	72.9	68.1
	**NOM**			64	32 (50.0)	32 (50.0)				
**CTLA-4_var2**	**OSCC**	0.001	9.14	102	93 (91.2)	9 (8.8)	91.2	60.3	78.8	80.9
	**NOM**			63	25 (39.7)	38 (60.3)				
**CD80**	**OSCC**	0.001	9.04	99	79 (79.8)	20 (20.2)	79.8	86.5	91.9	69.2
	**NOM**			52	7 (13.5)	45 (86.5)				
**CD86_Iso1**	**OSCC**	0.001	5.84	104	75 (72.1)	29 (27.9)	72.1	56.9	72.8	56.1
	**NOM**			65	28 (43.1)	37 (56.9)				

**Table 5:** **Correlation between the expression of the immune checkpoints CTLA-4 and CD28 and their ligands CD80 and CD86 in the total sample**. For the correlation analysis, all samples were included (OSCC and NOM) and Spearman’s correlations were calculated. Spearman’s ρ was used to describe the strength of correlation. Statistically relevant results are shown in bold.

		CTLA-4_var1	CTLA-4_var2	CD28	CD80	CD86_Iso1
**CTLA-4_var2**	Spearman’s ρ	**0.838**				
	*p*-value	**<0.001**				
	N	160				
**CD28**	Spearman’s ρ	**0.772**	**0.759**			
	*p*-value	**<0.001**	**<0.001**			
	N	157	156			
**CD80**	Spearman’s ρ	**0.388**	**0.463**	**0.234**		
	*p*-value	**<0.001**	**<0.001**	**0.008**		
	N	131	133	129		
**CD86_Iso1**	Spearman’s ρ	**0.845**	**0.755**	**0.748**	**0.352**	
	*p*-value	**<0.001**	**<0.001**	**<0.001**	**<0.001**	
	N	164	162	160	133	
**CD86_Iso3**	Spearman’s ρ	**0.648**	**0.514**	**0.626**	0.174	**0.733**
	*p*-value	**<0.001**	**<0.001**	**<0.001**	0.062	**<0.001**
	N	146	144	146	116	147

## Data Availability

The datasets presented in this article are not readily available because the data are part of an ongoing study. Requests for access should be directed to the corresponding author.
